# Loss of diacylglycerol kinase **ε** causes thrombotic microangiopathy by impairing endothelial VEGFA signaling

**DOI:** 10.1172/jci.insight.146959

**Published:** 2021-05-10

**Authors:** Dingxiao Liu, Qiong Ding, Dao-Fu Dai, Biswajit Padhy, Manasa K. Nayak, Can Li, Madison Purvis, Heng Jin, Chang Shu, Anil K. Chauhan, Chou-Long Huang, Massimo Attanasio

**Affiliations:** 1 Department of Internal Medicine, University of Iowa, Iowa City, Iowa, USA.; 2 Department of Vascular Surgery, The Second Xiangya Hospital, Central South University, Changsha, China.; 3 Department of Pathology, University of Iowa, Iowa City, Iowa, USA.; 4 Department of Emergency Medicine, Tianjin Medical University General Hospital, Tianjin, China.

**Keywords:** Nephrology, Inositol phosphates, Microcirculation

## Abstract

Loss of function of the lipid kinase diacylglycerol kinase ε (DGKε), encoded by the gene *DGKE*, causes a form of atypical hemolytic uremic syndrome that is not related to abnormalities of the alternative pathway of the complement, by mechanisms that are not understood. By generating a potentially novel endothelial specific *Dgke*-knockout mouse, we demonstrate that loss of *Dgke* in the endothelium results in impaired signaling downstream of VEGFR2 due to cellular shortage of phosphatidylinositol 4,5-biphosphate. Mechanistically, we found that, in the absence of DGKε in the endothelium, Akt fails to be activated upon VEGFR2 stimulation, resulting in defective induction of the enzyme cyclooxygenase 2 and production of prostaglandin E_2_ (PGE_2_). Treating the endothelial specific *Dgke*-knockout mice with a stable PGE_2_ analog was sufficient to reverse the clinical manifestations of thrombotic microangiopathy and proteinuria, possibly by suppressing the expression of matrix metalloproteinase 2 through PGE_2_-dependent upregulation of the chemokine receptor CXCR4. Our study reveals a complex array of autocrine signaling events downstream of VEGFR2 that are mediated by PGE_2_, that control endothelial activation and thrombogenic state, and that result in abnormalities of the glomerular filtration barrier.

## Graphical abstract

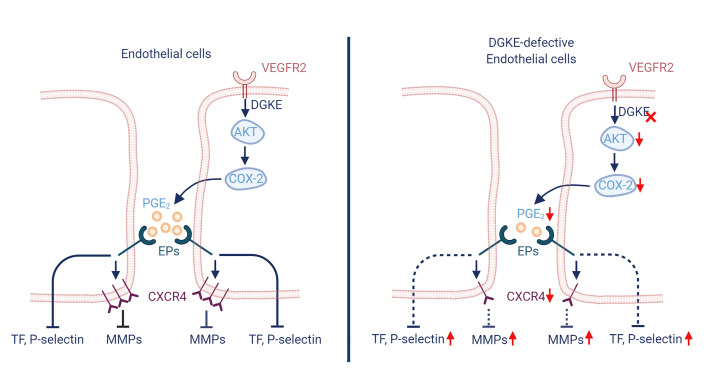

## Introduction

Atypical hemolytic uremic syndrome (aHUS) is a form of thrombotic microangiopathy (TMA) characterized by hemolytic anemia, thrombocytopenia, and acute renal failure not associated with enteric infections caused by Shiga toxin–producing bacteria. TMA in aHUS affects invariably the microvasculature in the renal glomeruli, causing renal insufficiency. Mutations in complement and complement-regulating genes have been identified in about 50% of idiopathic aHUS cases, pointing at the central role of the complement cascade in this disease (1). Notwithstanding, we and others described a genetic form of aHUS that is caused by mutations in the gene *DGKE* that encodes the lipid kinase diacylglycerol kinase ε (DGKε), revealing that factors unrelated to the complement system can cause aHUS by mechanisms that are not understood (2, 3).

**Figure 1:**
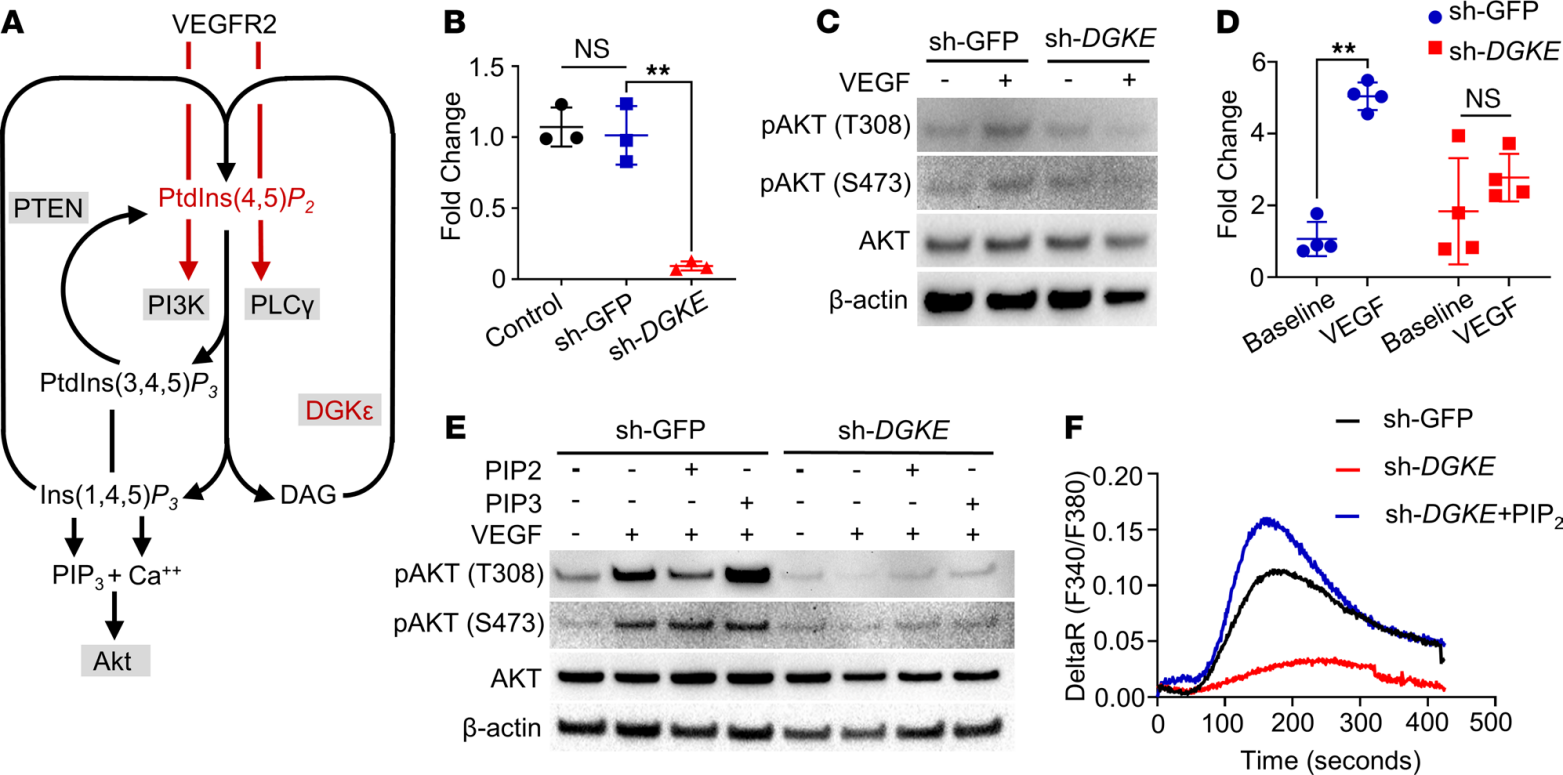
VEGFR2-dependent Akt activation is compromised in DGKE-knockdown human umbilical vein epithelial cells. (**A**) Schematic representation of the phosphoinositide cycle and of some of the enzymes involved in the cycle (boxes). Red arrows point to the major enzymes (PI3K and phospholipase C γ, PLCγ) activated downstream of VEGFR2. Black arrows represent generation of PtdIns(1,4,5) *P*_3_ (PIP3) and Ca^2+^ downstream of PI3K and PLCγ, respectively, and activation of Akt. The common substrate of PI3K and PLCγ, PIP2, required for Akt activation is highlighted in red. DGKε is highlighted in red. (**B**) Efficiency of the sh-RNA knockdown in HUVECs measured by quantitative PCR (Q-PCR) compared with nontargeted control (sh-GFP) cells. Control: nontransfected cells. (**C**) Western blot showing impaired Akt activation (phosphorylation of threonine 308 and serine 473) in the shRNA-knockdown HUVECs upon VEGFA stimulation, compared with nontargeted control cells. (**D**) Expression of Cox2, measured by Q-PCR, is not induced in DGKE-knockdown HUVECs compared with nontargeted control cells stimulated with VEGFA. (**E**) Western blots showing that impaired Akt activation in the sh-RNA knockdown HUVECs upon VEGFA stimulation is partially reversed by PIP2 and PIP3 supplementation. (**F**) Changes in intracellular Ca^2+^ concentrations after VEGFA supplementation in DGKE-knockdown HUVECs, in DGKE-knockdown HUVECs after PIP2 supplementation, and in nontargeted HUVEC controls over time, measured by Fura-2 AM fluorescence. Data are from 3–4 independent experiments and are presented as mean ± SD. **: *P* < 0.01 by 1-way ANOVA in **B** and by Student’s *t* test in **D**. Each data point represents 1 experiment. PTEN, phosphatase and tensin homolog; pAKT, phosphorylated Akt; DeltaR: fluorescence ratio as 340 nm/380 nm.

DGKε is a lipid kinase with high specificity for diacylglycerol (DAG) conjugated with arachidonic acid (AA) at the second carbonyl group of DAG (4–7). As an effect of the DGKε activity, cellular arachidonoyl poly-phosphatidyl-inositides (PtdIns) predominate over PtdIns acylated with other acyl moieties, and, as a consequence, in *Dgke*-knockout cells the total amount of PtdIns is 3-fold or lower than in WT cells (4–8). Under basal conditions phosphatidyl-inositol diphosphate [PtdIns(4,5) *P*_2_, henceforth PIP2] is the most abundant cellular PtdIns. Importantly, it is consumed upon activation of multiple receptors, including receptor tyrosine kinases such as VEGFR2, and it needs to be regenerated continuously through the phosphoinositide cycle (Figure 1A) (9–12).

Here, based on in vitro studies on primary endothelial cells and on the use of an endothelial specific *Dgke*-knockout mouse, we show that the signaling downstream of VEGFR2 is compromised in the absence of DGKε in the endothelium because of impaired activation of Akt and that the latter is restored by increasing cellular levels of PIP2. Mechanistically, we found that defective Akt activation in endothelial cells results in lack of expression of the gene prostaglandin-endoperoxide synthase 2, which encodes the enzyme cyclooxygenase 2 (Cox2) and induces the synthesis of its main product prostaglandin E_2_ (PGE_2_). Treating endothelial specific *Dgke*-knockout mice with a stable PGE_2_ analog reversed not only the aHUS, but also the proteinuria, possibly through CXCR4-mediated downregulation of matrix metalloproteinase 2 (MMP-2). Our data indicate that the endothelium is the tissue that is primarily affected by loss of *Dgke* and reveal multiple complex autocrine signaling events downstream of VEGFR2 that result in endothelial activation, thrombogenic state, and abnormalities of the glomerular filtration barrier. These signaling steps may be manipulated to modify the clinical course of the *DGKE*-related aHUS and conceivably of other diseases caused by endothelial activation or abnormal angiogenesis.

## Results

### VEGFR2-dependent Akt activation is compromised in DGKE-knockdown human umbilical vein endothelial cells and in human microvascular endothelial cells.

VEGFR2 is expressed in endothelial cells and exerts proproliferative, prosurvival, and promigratory functions, promoting angiogenesis upon stimulation by VEGFA (13–17). VEGFR2 signals downstream by autophosphorylating multiple tyrosine residues, where effector proteins are recruited (14). This results in, among other effects, activation of PLCγ, which then hydrolyzes PIP2 into inositol 1,4,5-triphosphate [Ins(1,4,5)*P*_3_, hereafter IP3] and DAG, and activation of phosphatidylinositol-3-kinase (PI3K), which leads to the generation of PIP3 from its precursor, PIP2, and ultimately to the activation of Akt (Figure 1A) (18–22). Since VEGF signaling is crucial to the maintenance of the glomerular microvasculature (23, 24), and because the disruption of VEGF signaling in humans and mice results in glomerular lesions that resemble those observed in humans with loss-of-function mutations in *DGKE* (2, 25), we hypothesized that the loss of *DGKE* may impair VEGF signaling in endothelial cells because of the shortage of PIP2 (Figure 1A). To test this hypothesis, we knocked down *DGKE* in human umbilical vein endothelial cells (HUVECs) by shRNA interference, using a nontargeting construct (sh-GFP) as a control (Figure 1B), and tested the activation of Akt by Western blot (phosphorylation of threonine 308 and serine 473 of Akt) upon VEGFA stimulation. Unlike untransfected and sh-GFP–transfected cells, *DGKE*-knockdown cells failed to activate Akt when exposed to VEGFA (Figure 1C and Supplemental Figure 1, A and B; supplemental material available online with this article; https://doi.org/10.1172/jci.insight.146959DS1). Consistent with the impaired Akt activation, the expression of Cox2, which in endothelial cells is induced by VEGFA (26–29), was also compromised (Figure 1D). However, Akt activation was partially rescued when cells were exposed to PIP2 and, as expected, to PIP3 (Figure 1E and Supplemental Figure 1, C and D). Likewise, intracellular calcium levels that are mobilized from the endoplasmic reticulum by increased intracellular concentrations of IP3 did not rise after VEGFA stimulation, but they did increase after supplementing the culture medium with PIP2 (Figure 1F). We obtained overlapping results using human microvascular endothelial cells (HMECs) (Supplemental Figure 2, A and B). These results indicate that Akt activation downstream of VEGFR2 is defective in *DGKE*-knockdown endothelial cells because of decreased availability of PIP2.

### Endothelial specific Dgke-knockout mice fully recapitulate the human disease.

**Figure 2:**
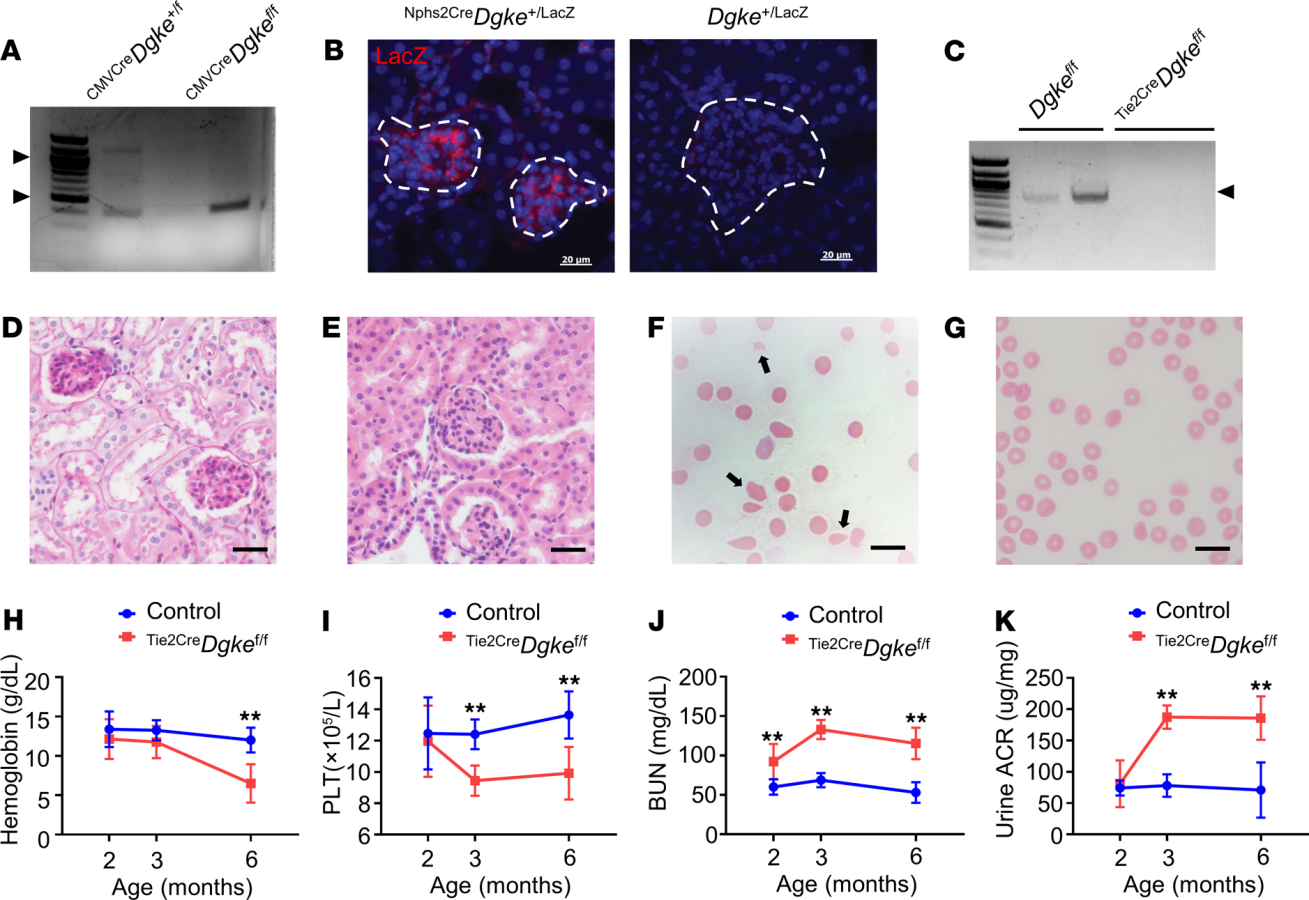
Endothelial specific Dgke-knockout mice recapitulate the full human phenotype. (**A**) Agarose gel of the PCR products of the amplification of genomic DNA from ubiquitously Cre-expressing ^CMVCre^*Dgke*^fl/fl^ mice compared with ^CMVCre^*Dgke*^+/fl^ heterozygotes. Heterozygous mice show a long band of 1201 bp corresponding to the retained exon 2 and a shorter band of 365 bp corresponding to the excised exon 2 (arrowheads). Exon 2 is not retained in the homozygous status. (**B**) Immunofluorescence microscopy images of glomeruli (dashed lines) from ^Nphs2Cre^*Dgke*^+/LacZ^ knockin mice compared with *Dgke*^+/LacZ^ controls, showing LacZ expression exclusively in glomeruli. Scale bars are 20 μm. (**C**) RT-PCR on RNA showing a band of 830 bp (arrowhead) in *Dgke*^fl/fl^ mice and no product in ^Tie2Cre^*Dgke*^fl/fl^ mice. Primers were placed in the corresponding exon 1 and 5 in the cDNA of *Dgke*. (**D**) Representative bright-field microscopy images of glomeruli of Periodic acid–Schiff–stained (PAS-stained) and (**E**) H&E-stained sections of ^Tie2Cre^*Dgke*^fl/fl^ mouse kidneys showing near-complete occlusion of the capillary tuft. Scale bars are 50 μm. (**F**) Smears of blood from ^Tie2Cre^*Dgke*^fl/fl^ and (**G**) *Dgke*^fl/fl^ controls at 6 months of age. Numerous schistocytes (arrows) are present in ^Tie2Cre^*Dgke*^fl/fl^ knockouts. Scale bars are 75 μm. (**H**–**K**) Serum hemoglobin, circulating platelets (PLT), blood urea nitrogen (BUN), and urine albumin to creatinine ratio (ACR) in ^Tie2Cre^*Dgke*^fl/fl^ compared with *Dgke*^fl/fl^ controls at 2, 3, and 6 months of age. Data are presented as mean ± SD. **: *P* < 0.01 by Student’s *t* test. *n* = 6 mice per group.

To test if VEGFA signaling in endothelial cells lacking Dgke is also compromised in vivo, we generated a conditional knockout mouse in which exon 2 of *Dgke* was deleted in a tissue-specific manner upon Cre recombination, using knockout first embryonic stem (ES) cells from the Knockout Mouse Project (KOMP) repository (30, 31). C57BL/6 *Dgke*-floxed mice are viable and fertile, and they produce progeny at the expected Mendelian ratio. We confirmed that exon 2 of *Dgke* is excised upon Cre recombination, by using the CMV-Cre mice to delete *Dgke* in all tissues (32) (Figure 2A). We also verified that the expression of the transgene is tissue specific, by generating ^Nphs2Cre^*Dgke*^LacZ^ knockin mice and showing that the LacZ expression was present exclusively in the glomeruli (30, 33) (Figure 2B). To generate endothelial specific *Dgke*-knockout mice, *Dgke*-floxed mice were then crossed to mice in which the Cre recombinase is driven by the angiopoietin receptor Tie2 promoter in endothelial cells and in 2% to 7% of blood mononuclear cells (34). We assessed the knockout efficiency by real-time PCR (RT-PCR) on RNA extracted from ^Tie2Cre^*Dgke*^fl/fl^ lungs, which is one of the most vascularized tissues (Figure 2C), given that no antibody is available that reliably detects Dgke. We excluded the possibility that abnormal platelet function due to expression of Tie2 in megakaryocytes could contribute to the observed phenotype by performing in vitro studies of platelet aggregation (Supplemental Figure 3). Unlike the constitutive *Dgke*-knockout mice that do not have spontaneous phenotype, endothelial specific ^Tie2Cre^*Dgke*^fl/fl^ conditional knockout mice developed occlusion of the glomerular capillaries (Figure 2, D and E), schistocytosis (Figure 2, F and G, and Supplemental Figure 4A), hemolytic anemia (Figure 2H and Supplemental Figure 4B), thrombocytopenia (Figure 2I), and renal insufficiency as early as 2 months of age (Figure 2J). Remarkably, these mice also developed albuminuria (Figure 2K), which is observed in all patients affected by DGKE disease, by 3 months of age. The presence of albuminuria in endothelial specific ^Tie2Cre^*Dgke*^fl/fl^ knockout mice and its appearance at a later time, compared with the renal insufficiency, suggest that the endothelium is the cellular compartment responsible for the DGKE disease and that the impairment of the glomerular barrier may be a later and secondary event. Interestingly, we did not detect obvious signs of TMA in liver, small intestine, and spleen arterioles of ^Tie2Cre^*Dgke*^fl/fl^ knockout mice (Supplemental Figure 5), which may indicate a particular susceptibility of the glomerular microvasculature to this disease.

### A phosphatase and tensin homolog inhibitor rescues the phenotype of endothelial specific Dgke-knockout mice.

**Figure 3:**
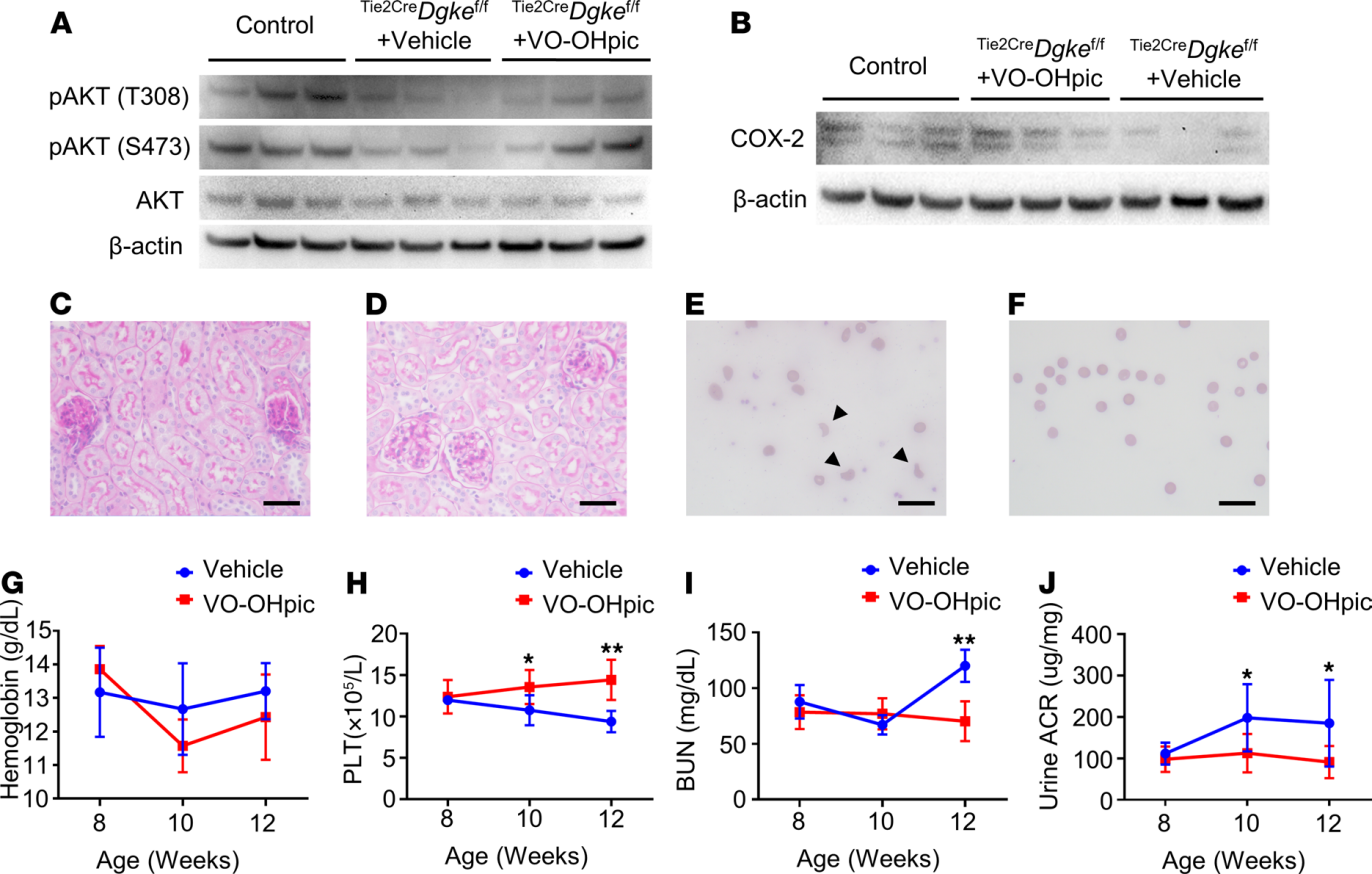
A PTEN inhibitor rescues the phenotype of endothelial-specific Dgke-knockout mice. (**A**) Western blot showing impaired Akt activation (phosphorylation of threonine 308 and serine 473) in kidney cortex extracts of ^Tie2Cre^*Dgke*^fl/fl^ mice compared with controls. Akt activation was partially rescued in VO-OHpic–treated ^Tie2Cre^*Dgke*^fl/fl^ littermates. (**B**) Western blot showing increased protein levels of COX2 in kidney cortex extracts of VO-OHpic–treated ^Tie2Cre^*Dgke*^fl/fl^ mice compared with ^Tie2Cre^*Dgke*^fl/fl^ littermates. (**C**) Representative bright-field microscopy images of glomeruli of PAS-stained ^Tie2Cre^*Dgke*^fl/fl^ mouse kidneys and (**D**) ^Tie2Cre^*Dgke*^fl/fl^ littermates’ kidneys after 4 weeks of treatment with the PTEN inhibitor VO-OHpic. The occlusion of the glomerular capillaries is rescued after VO-OHpic treatment. Scale bars are 50 μm. (**E**) Smears of blood from ^Tie2Cre^*Dgke*^fl/fl^ mice and (**F**) ^Tie2Cre^*Dgke*^fl/fl^ littermates after VO-OHpic treatment at 3 months of age. Schistocytes are found in ^Tie2Cre^*Dgke*^fl/fl^ mice (arrowheads) but not in VO-OHpic–treated mice. Scale bars are 50 μm. (**G**–**J**) Serum hemoglobin, circulating PLT, BUN, and urine ACR in ^Tie2Cre^*Dgke*^fl/fl^ mice compared with VO-OHpic–treated ^Tie2Cre^*Dgke*^fl/fl^ littermates at 8, 10, and 12 weeks of age. Data are presented as mean ± SD. *: *P* < 0.05, **: *P* < 0.01 by Student’s *t* test. *n* = 6 mice per group.

As observed in the *DGKE*-knockdown cells, Akt phosphorylation was lower in kidney cortexes of ^Tie2Cre^*Dgke*^fl/fl^ mice, compared with controls (Figure 3A, lanes 1 through 6; and Supplemental Figure 1, E and F). Hence, we tested whether increasing the endothelial levels of PIP3 would rescue the phenotype in vivo. To this end, we crossed ^Tie2Cre^*Dgke*^fl/fl^ mice to *Pten*-floxed mice (35), to obtain ^Tie2Cre^*Dgke*^fl/fl^ *Pten*^+/fl^ in which only 1 of the 2 *Pten* alleles is present in endothelial cells; however, we were not able to obtain pups of the desired genotype, likely because of in utero mortality. For this reason, we used a potent small molecule inhibitor of PTEN (VO-OHpic) (36), and injected 8-week-old ^Tie2Cre^*Dgke*^fl/fl^ mice i.p. daily for 4 weeks. As expected, treating the ^Tie2Cre^*Dgke*^fl/fl^ mice with the PTEN inhibitor resulted in increased Akt activation in renal cortexes (Figure 3A, lanes 4 through 9; and Supplemental Figure 1, E and F). Consistent with Akt activation, the expression of Cox2 in kidney cortexes was increased in ^Tie2Cre^*Dgke*^fl/fl^ mice treated with PTEN inhibitor compared with vehicle-treated ^Tie2Cre^*Dgke*^fl/fl^ mice (Figure 3B and Supplemental Figure 1G). Remarkably, VO-OHpic–treated mice were protected from developing glomerular capillary obstruction (Figure 3, C and D), thrombocytopenia (Figure 3H), renal insufficiency, and proteinuria (Figure 3, I and J). Although schistocytosis was clearly evident in peripheral blood smears of vehicle-treated mice (Figure 3, E and F, and Supplemental Figure 4C), the serum hemoglobin concentrations did not significantly differ from those of VO-OHpic–treated mice (Figure 3G). These results indicate that Akt activation is impaired in kidneys of endothelial specific knockout mice and that increasing PIP3 cellular levels is sufficient to rescue the symptoms in ^Tie2Cre^*Dgke*^fl/fl^ knockout mice.

**Figure 4:**
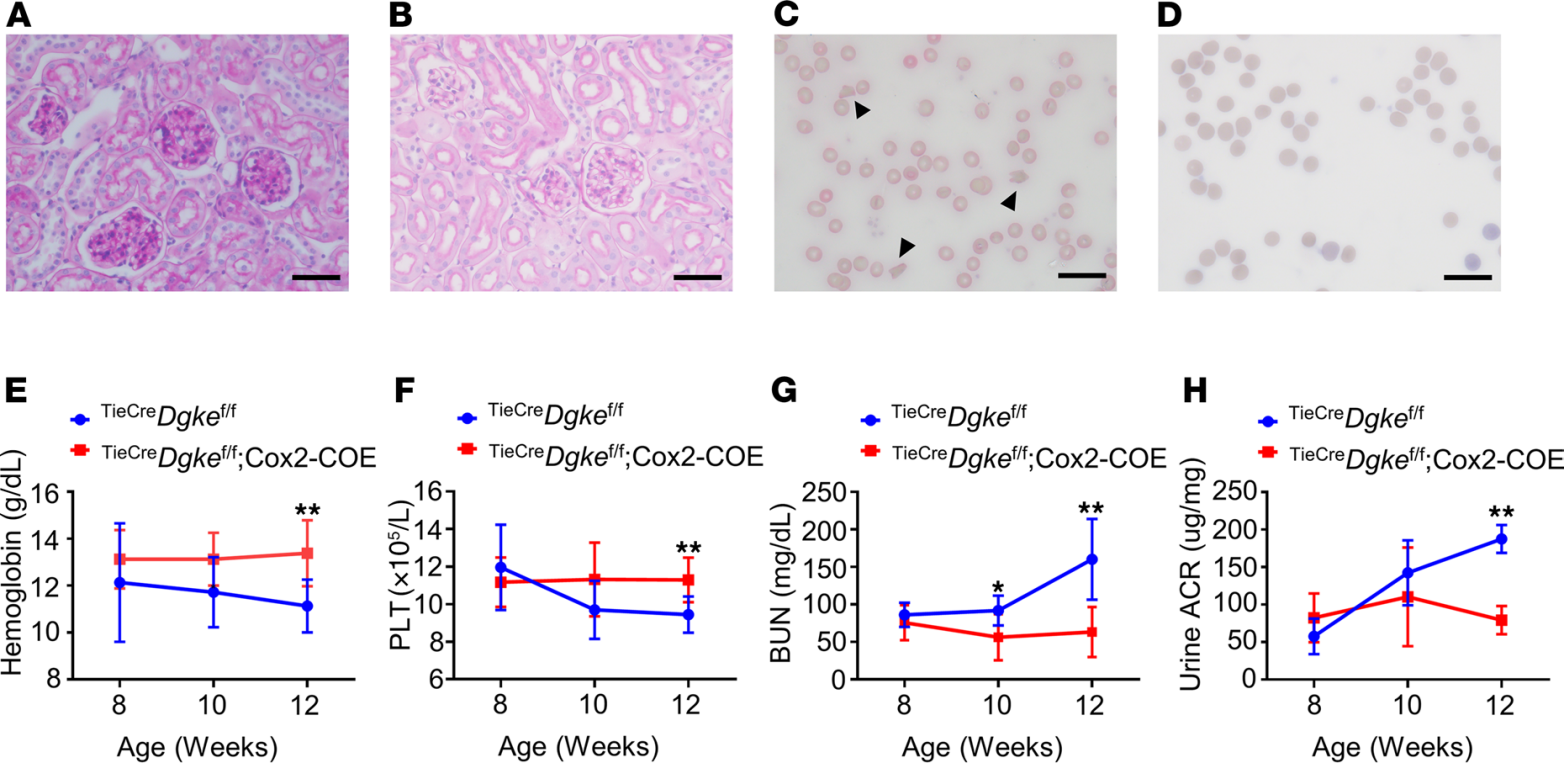
Overexpression of Cox2 in endothelial cells rescues the phenotype of endothelial specific Dgke-knockout mice. (**A**) Representative bright-field microscopy images of glomeruli of PAS-stained ^Tie2Cre^*Dgke*^fl/fl^ mouse kidneys and (**B**) ^Tie2Cre^*Dgke*^fl/fl^ Cox2-COE mouse kidneys at 12 weeks of age. The occlusion of the glomerular capillaries is rescued in ^Tie2Cre^*Dgke*^fl/fl^ Cox2-COE mice. (**C**) Smears of blood from ^Tie2Cre^*Dgke*^fl/fl^ knockout mice and (**D**) ^Tie2Cre^*Dgke*^fl/fl^ Cox2-COE mice at 3 months of age. Schistocytes are visible in ^Tie2Cre^*Dgke*^fl/fl^ knockout mice (arrowheads) and are absent in ^Tie2Cre^*Dgke*^fl/fl^ Cox2-COE mice. Scale bars are 50 μm. (**E**–**H**) Serum hemoglobin, circulating PLT, BUN, and urine ACR in ^Tie2Cre^*Dgke*^fl/fl^ and ^Tie2Cre^*Dgke*^fl/fl^ Cox2-COE mice at 8, 10, and 12 weeks of age. Data are presented as mean ± SD. *: *P* < 0.05, **: *P* < 0.01 by Student’s *t* test. *n* = 6 mice per group.

*Overexpression of Cox2 in endothelial cells rescues the phenotype of*^Tie2Cre^*Dgke*^fl/fl^*mice*. We previously reported that the expression of the gene prostaglandin-endoperoxide synthase 2, also known as cyclooxygenase 2 (*Cox2*), and the synthesis of its main product PGE_2_ were impaired in *Dgke* constitutive knockout mice (37). However, still undetermined is if impaired Cox2 induction is only associated with or determines the phenotype in *Dgke*-knockout mice. To answer this question, we crossed ^Tie2Cre^*Dgke*^fl/fl^ mice with B6.129S4-Tg(CAG-EGFP,-Ptgs2,-hrluc) (Cox2-COE) mice to obtain ^Tie2Cre^*Dgke*^fl/fl^ Cox2-COE double transgenics. Cox2-COE mice were engineered to carry a transgene that contains a CAG promoter that drives the expression of *Cox2*. The promoter is followed by a *loxP*-flanked sequence containing enhanced GFP and a transcriptional/translational STOP sequence that allows tissue-specific overexpression of Cox2 in the presence of Cre recombinase (38). As observed in ^Tie2Cre^*Dgke*^fl/fl^ mice treated with the PTEN inhibitor, ^Tie2Cre^*Dgke*^fl/fl^ Cox2-COE mice did not develop glomerular capillary occlusion (Figure 4, A and B), schistocytosis and hemolytic anemia (Figure 4, C–E, and Supplemental Figure 4D), thrombocytopenia (Figure 4F), and renal insufficiency and proteinuria (Figure 4, G and H). These results show that the lack of endothelial expression of *Cox2* is causative of the Dgke phenotype. They also denote that the failed induction of Cox2 in endothelial cells and not the lack of its substrate AA causes aHUS and proteinuria in ^Tie2Cre^*Dgke*^fl/fl^ mice.

### A stable PGE_2_ analog rescues the phenotype of endothelial specific Dgke-knockout mice.

**Figure 5:**
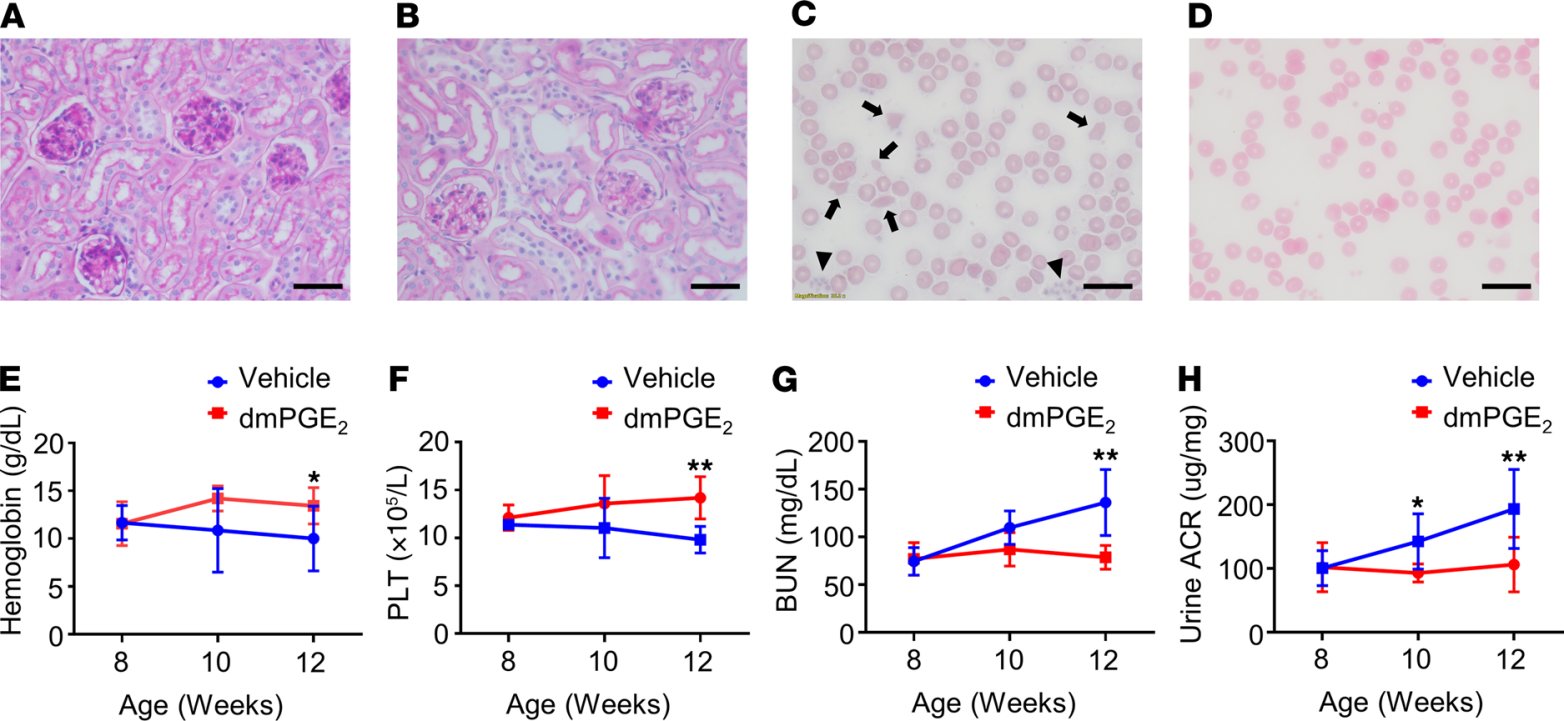
A stable PGE_2_-analog rescues the phenotype of ^Tie2Cre^Dgke^fl/fl^ mice. (**A**) Representative bright-field microscopy images of glomeruli of PAS-stained ^Tie2Cre^*Dgke*^fl/fl^ mouse kidneys and (**B**) ^Tie2Cre^*Dgke*^fl/fl^ mouse kidneys after 4 weeks of subcutaneous infusion of the PGE_2_ stable analog dmPGE_2_. The occlusion of the glomerular capillaries is rescued after the dmPGE_2_ treatment. Scale bars are 50 μm. (**C**) Smears of blood from ^Tie2Cre^*Dgke*^fl/fl^ knockout mice and (**D**) ^Tie2Cre^*Dgke*^fl/fl^ mice after dmPGE_2_ treatment at 3 months of age. ^Tie2Cre^*Dgke*^fl/fl^ knockout mice show numerous schistocytes (arrows) and circulating reticulocytes (arrowheads). Scale bars are 50 μm. (**E**–**H**) Serum hemoglobin, circulating PLT, BUN, and urine ACR in ^Tie2Cre^*Dgke*^fl/fl^ compared with dmPGE_2_-treated ^Tie2Cre^*Dgke*^fl/fl^ mice at 8, 10, and 12 weeks of age. Data are presented as mean ± SD. *: *P* < 0.05, **: *P* < 0.01 by Student’s *t* test. *n* = 6 mice per group.

Cox2 is the rate-limiting enzyme for the generation of PGE_2_, a potent promoter of endothelial cell migration, survival, and angiogenesis (39, 40). VEGFA induces Cox2 and PGE_2_ in endothelial cells (26, 28, 29, 41), which suggests that its proangiogenic effect might be mediated at least partly by the production of PGE_2_ by mechanisms that are not completely understood (27, 42, 43). In a precedent work we showed that urinary concentration of PGE_2_, but not of the stable PGI_2_ metabolite 6-keto PGF_1α_, was reduced in the *Dgke* constitutive knockouts compared with control mice. *Dgke-*knockout mice showed defective vascularization of surgical sponges implanted subcutaneously, and this defect was rescued by injecting the sponges with PGE_2_ (44), which pointed to the existence of a defect of PGE_2_-mediated angiogenesis in these mice. To clarify the role of PGE_2_ in determining the clinical manifestations in ^Tie2Cre^*Dgke*^fl/fl^ mice, we tested if increasing circulating levels of PGE_2_ would rescue the hematologic and renal manifestations in our mouse model. To this end, we implanted subcutaneous osmotic pumps in 8-week-old ^Tie2Cre^*Dgke*^fl/fl^ mice to continuously deliver the stable PGE_2_ analog 16,16 dimethyl-PGE_2_ (dmPGE_2_) and looked for changes in the phenotype after 4 weeks. Infusion of dmPGE_2_ was sufficient to normalize all the clinical manifestations in ^Tie2Cre^*Dgke*^fl/fl^ mice (Figure 5, A–H, and Supplemental Figure 4E). These results indicate that impaired PGE_2_ production is a key factor in determining the presence of disease in the absence of Dgke and that the anemia observed in these mice is hemolytic and not caused by bone marrow failure.

### Treatment with a PGE_2_ stable analog normalizes endothelial cell activation and prothrombotic diathesis in ^Tie2Cre^Dgke^fl/fl^ mice.

**Figure 6:**
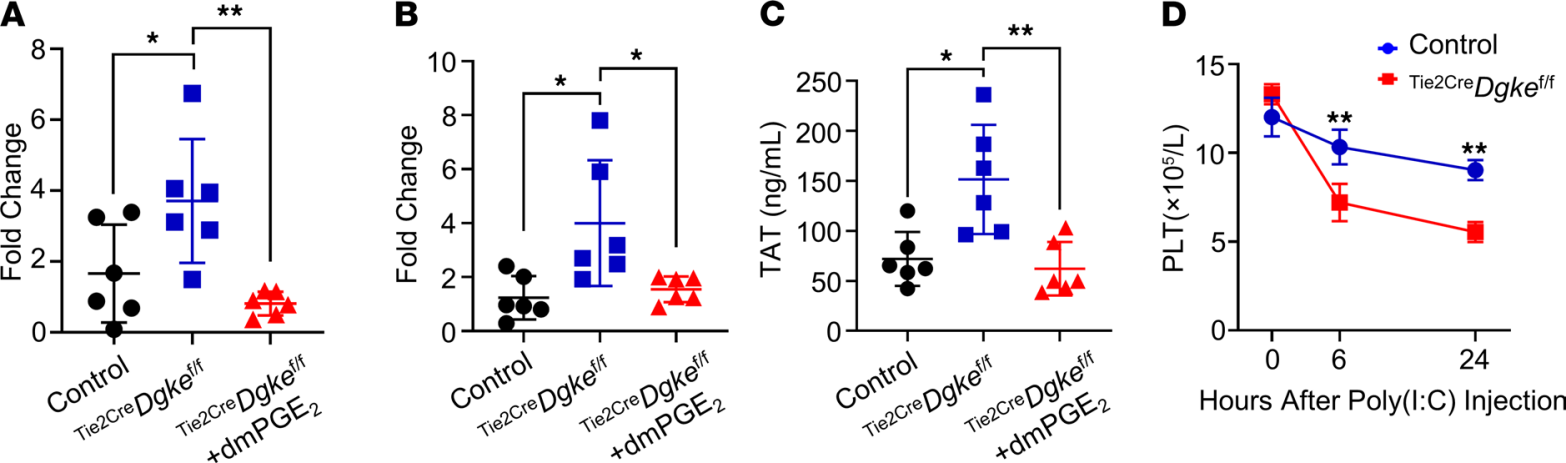
Treatment with a PGE_2_ stable analog normalizes endothelial cell activation and prothrombotic diathesis in ^Tie2Cre^Dgke^fl/fl^ mice. Q-PCR results showing increased (**A**) P selectin and (**B**) TF expression in kidney cortexes of ^Tie2Cre^*Dgke*^fl/fl^ mice compared with controls that were within normal range in ^Tie2Cre^*Dgke*^fl/fl^ littermates after 4 weeks of subcutaneous infusion of dmPGE_2_. (**C**) Thrombin-antithrombin complex (TAT) levels in plasma showing increased TAT levels in ^Tie2Cre^*Dgke*^fl/fl^ mice, compared with control mice. TAT levels were normalized after dmPGE_2_ infusion. (**D**) Counts of circulating PLT of ^Tie2Cre^*Dgke*^fl/fl^ mice and controls at 0, 6, and 24 hours after poly(I:C) injection. Data are presented as mean ± SD. *: *P* < 0.05, **: *P* < 0.01 by 1-way ANOVA in **A**–**C**, where each data point represents 1 mouse, and by Student’s *t* test in **D**; *n* = 6 mice per group.

*DGKE*-knockdown endothelial cells have been shown to express markers of activation and the initiator of coagulation tissue factor (TF) in vitro (45). We tested the expression of P selectin and TF in kidney cortexes of ^Tie2Cre^*Dgke*^fl/fl^ mice at 3 months of age and of plasma thrombin-antithrombin complexes (TAT) and found that all these parameters were significantly increased, compared with control mice (Figure 6, A–C), confirming in vivo in ^Tie2Cre^*Dgke*^fl/fl^ mice that lack Dgke have activation and prothrombotic state of the endothelium. In line with this observation, we also observed increased endothelial susceptibility to procoagulant inflammatory stimuli, by treating ^Tie2Cre^*Dgke*^fl/fl^ mice and controls at 2 months of age with the synthetic double-strand DNA polyinosinic:polycytidylic acid [poly(I:C)], a potent inducer of interferon-α (46) (Figure 6D). Of note, these results suggest that VEGFA signaling controls the activation and the thrombogenic state of the endothelium indirectly through the regulation of PGE_2_.

### PGE_2_ controls the endothelial expression of CXCR4 in the kidney.

**Figure 7:**
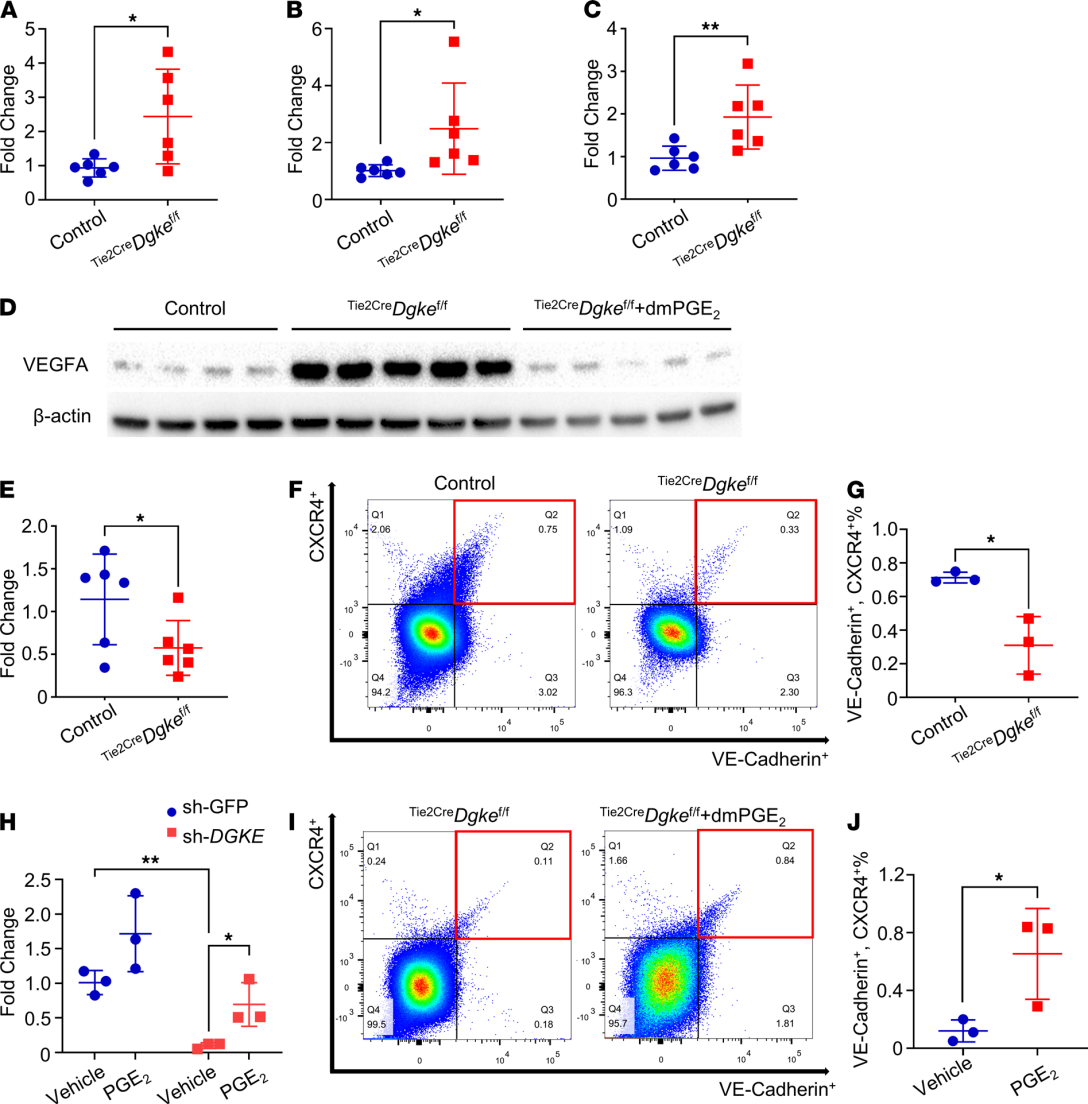
PGE_2_ controls the endothelial expression of CXCR4 in the kidney. Q-PCR showing increased (**A**) Hif1a, (**B**) Hif2a, and (**C**) Sdf-1 expression in kidney cortexes of ^Tie2Cre^*Dgke*^fl/fl^ mice compared with controls. (**D**) Western blot showing increased protein levels of VEGFA in kidney cortexes of ^Tie2Cre^*Dgke*^fl/fl^ mice compared with controls that were reversed in ^Tie2Cre^*Dgke*^fl/fl^ littermates after 4 weeks of subcutaneous infusion of dmPGE_2_. (**E**) Q-PCR showing impaired CXCR4 expression in kidney cortexes of ^Tie2Cre^*Dgke*^fl/fl^ mice compared with controls. (**F**) Representative flow cytometry plots and (**G**) statistical analysis of 3 independent flow cytometry experiments demonstrating decreased CXCR4^+^ endothelial cells (CXCR4^+^, VE-Cadherin^+^) in kidney cortexes of ^Tie2Cre^*Dgke*^fl/fl^ mice compared with controls. (**H**) mRNA expression of CXCR4 in sh-DGKE HUVECs and nontarget control cells (sh-GFP) with or without PGE_2_ supplementation showing that CXCR4 expression is rescued by dmPGE_2_. (**I**) Representative flow cytometry plots and (**J**) statistical analysis of 3 independent flow cytometry experiments showing decreased CXCR4^+^ endothelial cells (CXCR4^+^, VE-Cadherin^+^) in kidney cortexes from ^Tie2Cre^*Dgke*^fl/fl^ mice. CXCR4^+^, VE-Cadherin^+^ cells increased after 4 weeks’ infusion of dmPGE_2_ in ^Tie2Cre^*Dgke*^fl/fl^ littermates. Data are from 3 independent experiments and are presented as mean ± SD. **: *P* < 0.01, *: *P* < 0.05 by 1-way ANOVA in **H** and by Student’s *t* test in **A**–**C**, **E**, **G**, and **J**; *n* = 3–6 per group. Each data point represents 1 experiment or 1 mouse.

Stromal-derived factor 1 (SDF-1, also known as CXCL12) is a soluble chemokine expressed in the stromal component of several organs, and CXCR4 is its cognate receptor expressed on cells of the hematopoietic linage and on endothelial cells. In the kidney, like VEGFA, SDF-1 is expressed in podocytes (47–49). The activation of the SDF-1/CXCR4 signaling in endothelial cells exerts promigratory and proangiogenic effects, and it is required for the development of the renal vasculature (50). Hypoxia is a strong inducer of VEGFA, SDF-1, and CXCR4 (51, 52). In addition, high levels of SDF-1 are known to act in a feed-forward loop to further increase CXCR4 expression in endothelial cells (53). Because endothelial disfunction is expected to result in reduced tissue oxygenation, we tested the expression of hypoxia-induced genes in cortex extracts of ^Tie2Cre^*Dgke*^fl/fl^ and control mice and found that the expression of HIF-1 and HIF-2 was higher in ^Tie2Cre^*Dgke*^fl/fl^ kidney cortexes, compared with controls (Figure 7, A and B), as well as finding higher expression of HIF-1 and HIF-2 transcriptional targets SDF-1 and VEGFA (Figure 7, C and D). However, in spite of the hypoxic milieu of the ^Tie2Cre^*Dgke*^fl/fl^ kidney cortexes, the expression of CXCR4 was surprisingly low, compared with controls (Figure 7E). To confirm that the lower levels of CXCR4 in ^Tie2Cre^*Dgke*^fl/fl^ kidneys were caused by differences in CXCR4 expression in endothelial cells and not in other cell populations, we performed flow cytometry analysis of single-cell suspensions of kidney cortexes of ^Tie2-Cre^*Dgke*^fl/fl^ and control mice double-stained with antibodies against CXCR4 and against the endothelial cell marker VE-Cadherin. Consistent with other reports (54), VE-Cadherin^+^ endothelial cells represented about 3%–4% of the cells in our samples (Supplemental Figure 6B, Q2 + Q3). This analysis confirmed that, compared with controls, the proportion of CXCR4-expressing endothelial cells was reduced in ^Tie2-Cre^Dgke^fl/fl^ mice (Figure 7, F and G; and Supplemental Figure 6, A and C). Of note, because CXCR4 expression can be induced by PGE_2_ through the activation of adenylate cyclase (55–57), we tested the expression of *CXCR4* in *DGKE*-knockdown HUVECs and HMECs by Q-PCR and found that CXCR4 expression was strongly downregulated in these cells, but it was rescued to normal levels after supplementation with PGE_2_ (Figure 7H and Supplemental Figure 2C). Remarkably, treatment with dmPGE_2_ increased the number of endothelial cells expressing *CXCR4* in kidney cortexes of ^Tie2-Cre^*Dgke*^fl/fl^ mice (Figure 7, I and J). These results show that in the kidneys, PGE_2_ controls the endothelial expression of CXCR4 and thus the activity of the SDF-1/CXCR4 signaling axis.

### PGE_2_ suppresses the expression of MMP-2 in ^Tie2Cre^Dgke^fl/fl^ mouse kidneys.

**Figure 8:**
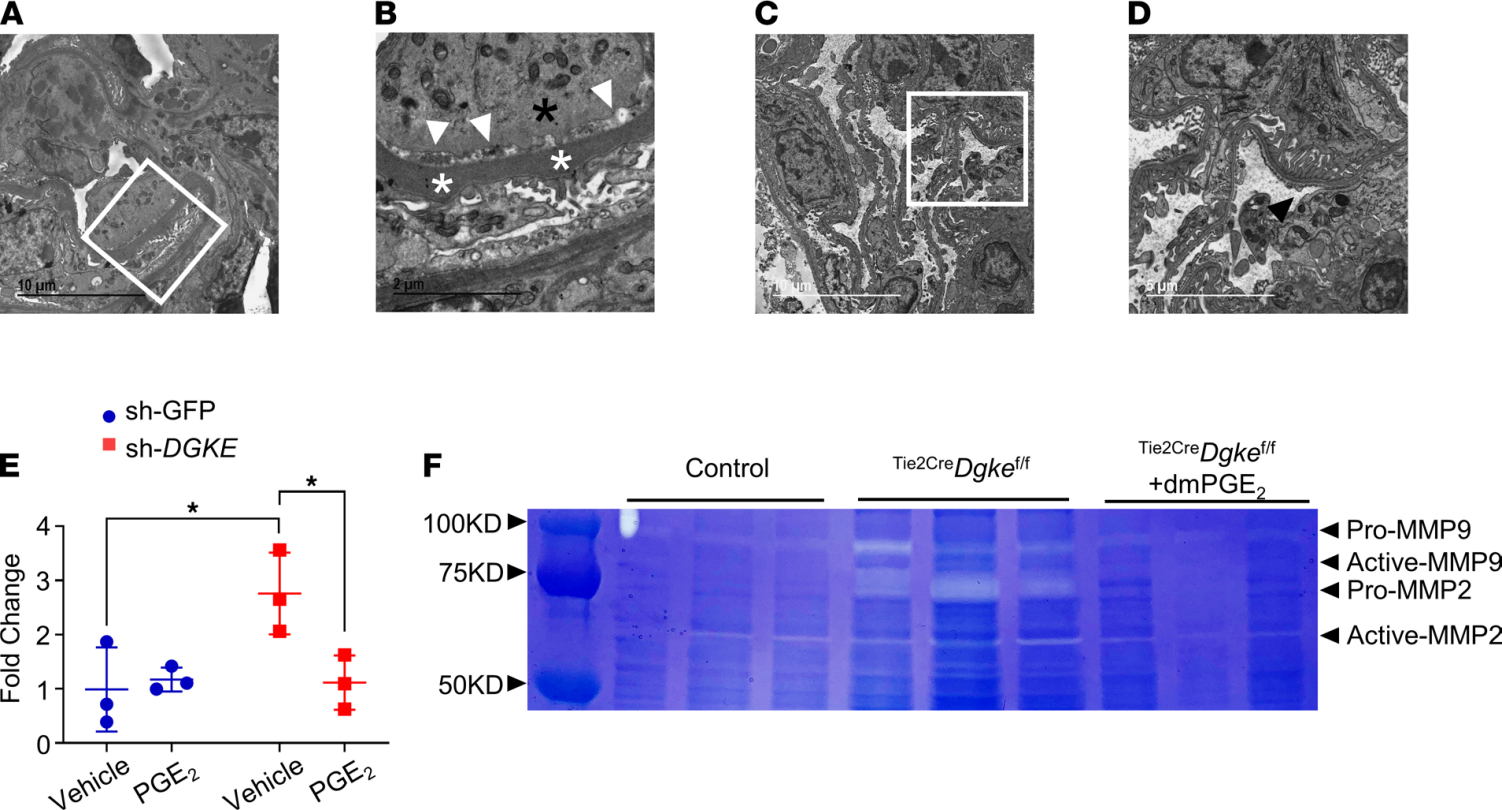
PGE_2_ suppresses the expression of MMP-2 in ^Tie2Cre^Dgke^fl/fl^ mouse kidneys. (**A** and **B**) Representative TEM images of a glomerulus of a 3-month-old ^Tie2Cre^*Dgke*^fl/fl^ mouse. (**B**) Higher magnification of the inset in **A**. White arrowheads point at subendothelial widening at the base of a swollen endothelial cell (black asterisk). White asterisks denote thickened GBM; black arrows, effaced foot processes. Scale bars: 10 μm (**A**), 2 μm (**B**). (**C** and **D**) Representative TEM image of a glomerulus of a 3-month-old ^Tie2Cre^*Dgke*^fl/fl^ mouse after 4 weeks’ subcutaneous infusion with dmPGE_2_. (**D**) Higher magnification of the inset in **C**. Black arrow points to normal foot processes; black arrowhead, healthy fenestrated endothelium and normal GBM. Scale bars: 10 μm (**C**), 5 μm (**D**). (**E**) Q-PCR showing MMP-2 expression in sh-*DGKE*–knockdown HUVECs and nontarget control cells (sh-GFP) before and after supplementation of the culture medium with PGE_2_. Data are from 3 independent experiments and are presented as mean ± SD. *: *P* < 0.05 by 1-way ANOVA, *n* = 3 per group. Each data point represents 1 experiment. (**F**) Gelatin-polyacrylamide gel showing the zymographic analysis of kidney cortex lysates of ^Tie2Cre^*Dgke*^fl/fl^ littermates of the same age after 4 weeks of dmPGE_2_ administration. Both pro–MMP-2 (72 kDa) and active MMP-2 (~60 kDa) are present in the ^Tie2Cre^*Dgke*^fl/fl^ mouse kidney, compared with controls, but disappear after treatment with dmPGE_2_.

DGKE nephropathy is invariably associated with proteinuria (3, 25, 58). Proteinuria was also present in 100% of endothelial specific ^Tie2-Cre^*Dgke*^fl/fl^ knockout mice at a later time compared with the appearance of the hematologic signs, which suggests that the filtration barrier is secondarily affected by primary endothelial abnormalities. To gain insights into the pathogenesis of the proteinuria in the ^Tie2-Cre^*Dgke*^fl/fl^ knockout mice, we performed transmission electron microscopy (TEM) of renal cortexes of these mice at 3 months of age, when albuminuria is detectable. ^Tie2-Cre^*Dgke*^fl/fl^ knockout mouse glomeruli showed moderate podocyte foot process effacement, subendothelial widening, and, interestingly, segmental thickening of the glomerular basement membrane (GBM) (Figure 8, A and B), but all these abnormalities were not detectable in dmPGE_2_-treated ^Tie2-Cre^*Dgke*^fl/fl^ mice, in concordance with the normalized albuminuria (Figure 8, C and D, and Figure 5F). For this reason, we investigated if the proteinuria in ^Tie2-Cre^*Dgke*^fl/fl^ mice could result from abnormalities of the GBM induced by the activated endothelium (59). In the bone marrow niche, SDF-1 and CXCR4 are essential regulators of the homing of hematopoietic progenitor cells (HPCs) (60–63), and inhibition of CXCR4 is used to force HPCs to egress from their niche into the bloodstream, when large numbers of HPCs need to be harvested for bone marrow transplantation (64). The mobilizing effect of CXCR4 inhibitors and other hematopoietic cell mobilizers is mediated by the induction of several proteinases, including MMPs (65). Considering that the expression of CXCR4 in endothelial cells can be induced by PGE_2_ (55–57), we tested by Q-PCR if impaired endothelial induction of PGE_2_ in the absence of *Dgke* would be indirectly associated with increased expression of MMP-2 and MMP-9, the MMPs most abundantly expressed in the glomeruli (66), compared with controls. We found that the expression of MMP-2 was increased in *DGKE*-knockdown HUVECs and HMECs and normalized by supplementing the culture medium with PGE_2_ (Figure 8E and Supplemental Figure 2D). MMP-2, but not MMP-9, expression was also higher in cortex extracts of ^Tie2-Cre^*Dgke*^fl/fl^ mice in vivo, but not in kidneys of mice 28 days after they were injured by folic acid injection (67) (Supplemental Figure 7). We further confirmed by gel zymography analysis of cortex lysates that the total MMP-2 protein levels and its proteolytic activity were increased in ^Tie2-Cre^*Dgke*^fl/fl^ mice but not in ^Tie2-Cre^*Dgke*^fl/fl^ mice treated with dmPGE_2_ (Figure 8F). These results indicate that MMP-2 activity is high in endothelial specific ^Tie2-Cre^*Dgke*^fl/fl^ mouse kidneys and suggest that chronic impairment of the VEGF signaling in these mice may favor the remodeling of the GBM, maybe by controlling PGE_2_ and CXCR4-dependent MMP-2 expression. Further studies that are beyond the scope of this work will be needed to further explore this possibility.

## Discussion

In this work we have investigated the molecular mechanisms by which biallelic loss-of-function mutations in *DGKE* cause a form of TMA and aHUS that it is not associated with defects of the alternative complement pathway (2, 3). By generating a potentially novel conditional knockout mouse for the gene *Dgke*, we demonstrated that endothelial specific deletion of *Dgke* is sufficient to phenocopy in full the human disease, which is remarkably similar to the disease reported in subjects treated with bevacizumab (2), highlighting the centrality of the endothelium in the DGKE disorder and suggesting that podocytes, which also express *DGKE*, are only secondarily affected. Mechanistically, we found that the shortage of PIP2, the intracellular substrate of PI3K, is the primary biochemical defect that causes the DGKE disease resulting in defective PIP3-dependent Akt activation in endothelial cells downstream of VEGFR2, as demonstrated in vitro and in vivo by the experiments in which a PTEN inhibitor was administered to^Tie2-Cre^*Dgke*^fl/fl^ mice. Thus, our data point at a central role of a defect of the Akt signaling in the endothelium of ^Tie2-Cre^*Dgke*^fl/fl^ mice and speak against a potential role of increased PKC activity in determining the DGKE phenotype, although this possibility cannot be completely excluded. Consistent with our in vivo data is also the conclusion, recently reported in an in vitro study, that by causing shortage of PIP2 (the common cellular substrate of both PI3K and PLC) loss of Dgke does not affect DAG signaling (8).

A somewhat unexpected connection that emerges from our studies is the relation between endothelial VEGF and Cox2/PGE_2_ signaling in endothelial cells. Although we previously reported the defect of Cox2 expression and PGE_2_ production in the constitutive *Dgke*-knockout mice, it was not clear how these abnormalities would be connected with the observed phenotype and if their correction would have been able to rescue the disease in *Dgke*-defective mice. The in vitro and in vivo experiments that we have reported here show that Dgke is required in endothelial cells to induce the expression of Cox2 and the production of PGE_2_ downstream of VEGFR2 and to promote the maintenance of the kidney microvasculature. This conclusion is supported by the ability of the endothelial overexpression of Cox2 and of the subcutaneous infusion of dmPGE_2_ to rescue the hematologic and renal manifestations observed in ^Tie2-Cre^*Dgke*^fl/fl^ mice. To this point, it is worthwhile to notice that treatment with dmPGE_2_ both rescued capillary occlusion and renal function, which could be explained by a vasodilator effect of PGE_2_ on the glomerular microvasculature, and avoided the activation of the endothelial cells and the expression of prothrombotic molecules, suggesting that the antiactivating effect of VEGFA on endothelial cells is exerted through the production of PGE_2_.

Finally, the studies that we have presented here imply an unanticipated interaction between VEGF and SDF-1/CXCR4 signaling in the kidney. A hypothetical general model that could be drawn from our observations would suggest the existence of a balance between proangiogenic VEGF-PGE_2_–mediated signaling that would cause, among other responses, the GBM remodeling by activating MMPs and negative feedback sustained by the counteracting SDF-1/CXCR4 signaling triggered by PGE_2_. According to such a model, the lack of Dgke and PGE_2_ would impair CXCR4-dependent suppression of MMP-2 in endothelial cells, and it would eventually result in glomerular filtration barrier damage (Graphical abstract). Although further experimental evidence has to be produced to better define the interplay between PGE_2_ and CXCR4 expression in vivo, our results reveal the existence of complex autocrine signaling events downstream of VEGFR2 that could conceivably be manipulated to modify the clinical course of aHUS and of other diseases characterized by endothelial activation or abnormal angiogenesis.

## Methods

### Animal studies.

All mice were on a C57BL/6J background. The Cox2-COE mice were provided by Harvey R. Herschman, University of California, Los Angeles (Los Angeles, California, USA), and were previously described (38).

### Generation of Dgke conditional knockout mice.

These mice were generated by injecting ES cells with a knockout first construct, from the KOMP (https://www.komp.org) (30). The knockout first allele is initially a nonexpressive form, but it can be converted to a conditional allele, which carries 2 *LoxP* sites flanking exon 2 of *Dgke*, via Flp recombination. We confirmed by genomic sequencing that this exon was deleted after Cre recombination. *Dgke*-floxed mice are viable and fertile, and they produce progeny at the expected Mendelian ratio. Tie2-Cre mice were purchased from The Jackson Laboratory (stock 008863) and were previously described (68). CMV-Cre mice were purchased from The Jackson Laboratory (stock 006054). WT mice bred from the colony were used as controls, unless otherwise specified.

### Endothelial cell culture.

HUVECs and human dermal microvascular endothelial cells (HMEC-1) were obtained from the American Type Culture Collection. Monolayers were cultured in medium 199 (Gibco, Thermo Fisher Scientific) supplemented with 20% heat-inactivated FBS (Gibco, Thermo Fisher Scientific), endothelial cell growth supplement (0.03 mg/mL), heparin (0.1 mg/mL), penicillin (100 IU/mL), and streptomycin (100 μg/mL). HMEC-1 monolayers were cultured in MCDB131 (Gibco, Thermo Fisher Scientific) supplemented with 15% heat-inactivated FBS (Gibco, Thermo Fisher Scientific), endothelial cell growth supplement (20 μg/mL), hydrocortisone (1 μg/mL), and glutamine (10 mM) in a humidified incubator at 37°C and 5% CO_2_/95% air.

### Lentiviral infection and shRNA-mediated gene silencing.

*DGKE* and GFP-targeting shRNA lentiviral constructs were purchased from Thermo Fisher Scientific. The experiments were performed as previously described (69).

### In vitro stimulation of HUVECs and HMECs with VEGF, with and without phosphoinositides.

Human VEGF recombinant protein was purchased from Gibco (Thermo Fisher Scientific). For VEGF treatment experiments, endothelial cells were first starved for 6 hours, then incubated with 80 ng/mL VEGF or vehicle control (PBS) for 15 minutes before being used for Western blot or intracellular calcium measurements. For phosphoinositide rescue experiments, cells were first starved for 6 hours. PIP2 or PIP3 (Echelon) was mixed with the carrier histone H1 (Echelon) at a 1:1 ratio. After a brief water bath sonication for 15 seconds, the mixture was incubated at room temperature for 15 minutes. Then it was added to the cell culture for 1 hour at the final concentration of 2.5 μM. Cells were then supplemented with VEGF or vehicle (PBS) as described above.

### Western blots.

Tissues were lysed in lysis buffer (150 mM NaCl, 10 mM EDTA, 10 mM Tris pH 7.4, 1% Triton X-100, DTT, and 25 mM N-Ethylmaleimide) with protease inhibitor cocktail (Roche) and phosphatase inhibitor cocktail (Roche). Protein concentration was calculated by NanoDrop (Thermo Fisher Scientific). Proteins were separated with NuPAGE 4%–12% Bis-Tris Gel (Invitrogen, Thermo Fisher Scientific) at 160 V, then transferred to a nitrocellulose membrane for 90 minutes at 100 V at 4°C. The membrane was blocked for 120 minutes in TBS-Tween with 5% BSA at room temperature and incubated with antibodies against anti–p-Akt (threonine 308) (Cell Signaling Technologies, 4056), anti–p-Akt (serine 473) (Cell Signaling Technologies, 9271), anti-Akt (Cell Signaling Technologies, 9272), anti-COX2 (Cell Signaling Technologies, 12282), and anti–β-actin (Cell Signaling Technologies, 8457) overnight at a dilution of 1:1000. Secondary rabbit antibody (Cell Signaling Technologies, 7074) and secondary mouse antibody (Cell Signaling Technologies, 7076) were incubated for 80 minutes at a concentration of 1:10,000 at room temperature. For exposure, Western ECL Substrate (Bio-Rad) was used. Imaging was done with ChemiDoc XRS+ System with Image Lab Software (Bio-Rad). Western blot densitometry was performed using ImageJ Version 1.53g, 2020 (NIH).

### Measurement of intracellular calcium.

VEGF-stimulated intracellular calcium release was measured in HUVECs. Briefly, cells were washed in PBS and subsequently loaded with 4 μM Fura-2 AM (Thermo Fisher Scientific) in Tyrode’s solution (140 mM NaCl, 5 mM KCl, 1.2 mM CaCl_2_, 1 mM MgCl_2_, 0.33 mM NaH_2_PO_4_, 5.5 mM glucose, 10 mM HEPES at pH 7.4 with NaOH) for 45 minutes at 37°C. The cells were then washed in PBS, seeded onto poly-l-lysine–coated glass coverslips, and incubated in complete medium for 1 hour at 37°C. After incubation, coverslips were transferred to Ca^2+^-free Tyrode’s solution, and the fluorescence ratio of the cells was measured at 340 nm and 380 nm at 37°C using MetaFluor Imaging Software (Molecular Devices), after 40 seconds of baseline recording (ΔR: fluorescence ratio as 340 nm/380 nm).

### Quantitative RT-PCR.

Total RNA was extracted using the RNeasy Mini Kit (QIAGEN). RNA was reverse-transcribed using the iScript cDNA Synthesis Kit (Bio-Rad). RT-PCR was performed using the CFX Connect Real-Time PCR Detection System (Bio-Rad) and iTaq Universal SYBR Green Supermix (Bio-Rad). All RT-PCR experiments were performed in triplicate. The sequences of PCR primers (IDT) are provided in Supplemental Table 1.

### Measurement of BUN, plasma TAT, serum lactate dehydrogenase activity, urine albumin, and urine creatinine.

Blood samples were obtained from the retro-orbital plexus. BUN and plasma TAT were measured by ELISA (Abcam), according to the manufacturer’s instructions. Twenty-four-hour urine was collected using metabolic cages. Serum lactate dehydrogenase (LDH) activity was tested by LDH assay kit (MilliporeSigma). Urinary albumin concentration was determined using the Albuwell microalbumin ELISA (Exocell). Urinary creatinine concentration was determined using a P/ACE MDQ Capillary Electrophoresis System and photodiode detector (Beckman Coulter) at the Physiology Core of the O’Brien Center for Kidney Diseases in Dallas (70). ACR was calculated by dividing albumin concentration in milligrams by creatinine concentration in grams.

### Measurement of circulating platelets and serum hemoglobin.

Mouse blood samples were collected from the retro-orbital plexus using heparinized capillary tubes. After acquisition, samples were immediately processed for platelet counts and hemoglobin measurements on an Advia 120 Hematology system (Siemens Medical Solutions USA Inc.) to avoid clot formation.

### Mouse platelet isolation and in vitro platelet aggregation studies.

Mouse platelets were prepared as previously described (71, 72). Blood from anesthetized mice was drawn from the retro-orbital plexus and collected in 1.5 mL polypropylene tubes containing 300 μL of enoxaparin (0.3 mg/mL; Sanofi-Aventis). The blood was centrifuged at 100*g* for 5 minutes, and the platelet-rich plasma (PRP) was collected in a fresh tube. PRP (platelet count; 2 × 10^8^/mL) from WT and endothelial specific knockout DGKE was stirred (178 RCF) at 37°C for 2 minutes in a whole blood/optical lumi-aggregometer (Chrono-log, model 700–2) before the addition of agonists (collagen or ADP). Aggregation was measured as percentage change in light transmission, where 100% refers to transmittance through the blank sample (platelet-poor plasma).

### PGE_2_ infusion with subcutaneous osmotic minipumps.

Mice were treated with dmPGE_2_ (Cayman Chemical) and vehicle control through a subcutaneously implanted ALZET 1004 mini–osmotic pump (DURECT). Minipumps were loaded with 100 μL of dmPGE_2_ solution in sterile PBS. A release rate of 0.11 μL/h administered a total amount of 30 μg/kg dmPGE_2_ daily, a dose that resulted in elevated serum PGE_2_ without obvious adverse side effects, during the 28 days of the experimental setup.

### PTEN inhibitor experiments.

Littermate ^Tie2^*Dgke*^fl/fl^ mice were treated with daily i.p. injection of the PTEN inhibitor VO-OHpic and vehicle control. VO-OHpic (BioVision, 1801-5) was used at 10 mg/kg/d, administered in a 10% DMSO solution.

### Poly(I:C) injections.

Mice were treated with i.p. injections (1 μg/μL, 200 μg/mouse) of a solution of poly(I:C) (MilliporeSigma, P1530). Blood and urine samples were collected at 6 hours, 24 hours, and 7 days after. Platelets, BUN, and ACR were measured as described above.

### Flow cytometry.

Fresh mouse kidney cortexes were digested in 2 mL of protease solution — 5 mM CaCl_2_, 10 mg/mL Bacillus licheniformis protease (Creative Enzymes, NATE0633), and 125 U/mL DNase I (Roche) in Dulbecco’s PBS — on ice for 30 minutes. The lysates were filtered through 70 μm cell strainers. Single-cell suspension was collected by rinsing with FACS buffer. Suspensions were lysed with red blood cell lysis buffer (Quality Biological) to remove red blood cells. After washing with 1× PBS, 1 × 10^6^ cells were resuspended in 100 μL of FACS buffer and preincubated with anti–mouse CD16/32 antibody (BioLegend, 101302) for blocking prior to labeling with CD144-APC (BioLegend, 138012) and CD184-BV421 (BD Biosciences, 562738) antibodies for 30 minutes at room temperature. Cells were then washed with FACS buffer and analyzed on BD LSR II Violet at the University of Iowa Flow Cytometry Facility. All flow cytometry results were analyzed using FlowJo software (Version 9).

### Zymography.

Kidney cortical tissues were lysed in lysis buffer (20 mM Tris-HCl, 125 mM NaCl, and 1% Triton X-100, pH 8.5) with protease inhibitor cocktail (Roche). Protein concentration was calculated by NanoDrop (Thermo Fisher Scientific). Samples were separated on Tris-glycine gels with 0.1% gelatin (Invitrogen, Thermo Fisher Scientific). After electrophoresis, the gels were incubated in 1× renaturing buffer (Thermo Fisher Scientific) for 2 hours at room temperature, then in 1× developing buffer (Thermo Fisher Scientific) for 48 hours. Gels were stained with Coomassie Blue R-250 (Bio-Rad) and destained in 20% ethanol and 10% acetic acid. MMP-2 activity was detected as clear bands on the blue background.

### Statistics.

All quantitative data are presented as mean ± SD. Statistical significance (*P* ≤ 0.05 was considered significant) was calculated using the 2-tailed unpaired Student’s *t* test for 2 groups and 1-way ANOVA for multiple-group comparison by Prism 7 (GraphPad Software).

### Study approval.

All procedures were approved by the Institutional Animal Care and Use Committee of the University of Iowa (protocol 7021976).

## Author contributions

DL performed experiments, analyzed data, prepared figures, and contributed to writing the manuscript. QD, MP, BP, CL, MP, DFD, and MKN performed experiments and analyzed data. HJ, AKC, CS, and CLH provided reagents. MA designed experiments, analyzed data, prepared figures, and contributed to writing of the manuscript.

## Supplementary Material

Supplemental data

Supplemental Table 1

## Version 1 05/10/2021

Electronic publication
