# Supplementary material for: Loss of diacylglycerol kinase ε causes thrombotic microangiopathy by impairing endothelial VEGFA signaling
Source: JCI Insight. 2021 May 10;6(9):e146959. doi: 10.1172/jci.insight.146959 (PMC8262293; doi:10.1172/jci.insight.146959)
Supplement: Supplemental data [file jciinsight-6-146959-s018.pdf]

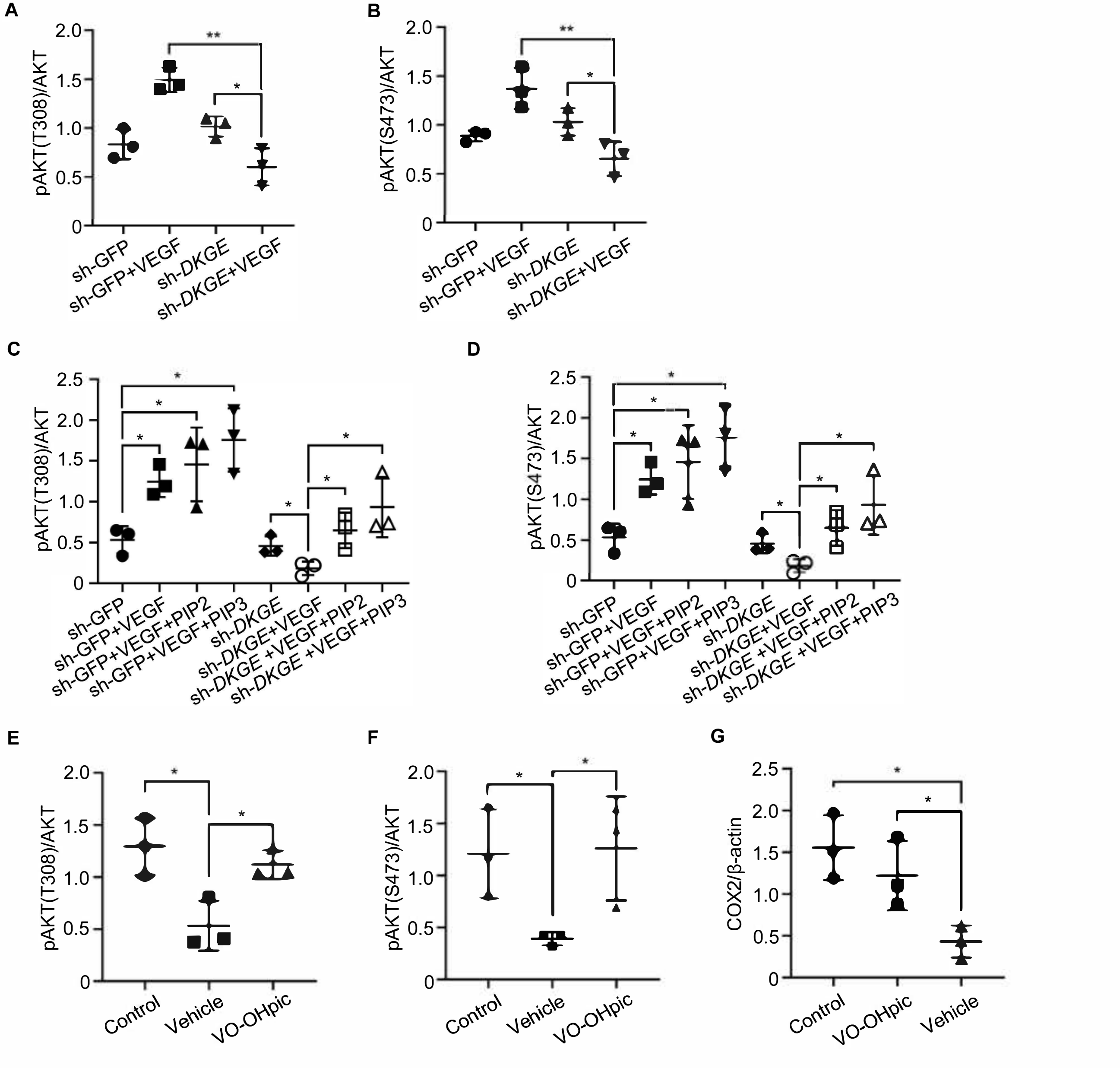

Supplementary figure 1. Densitometric analysis of the western blots presented in Figure 1C (**A-B**), in Figure 1E (**C-D**), Figure 3A (**E-F**) and Figure 3B (**G**). Total Akt was used as normalizer in panels A through F, β-actin was used in panel G. Data are presented as mean ±SD.*: *P*<0.05. **: *P*<0.01 (by Student's t-test). *n*=3 per group.

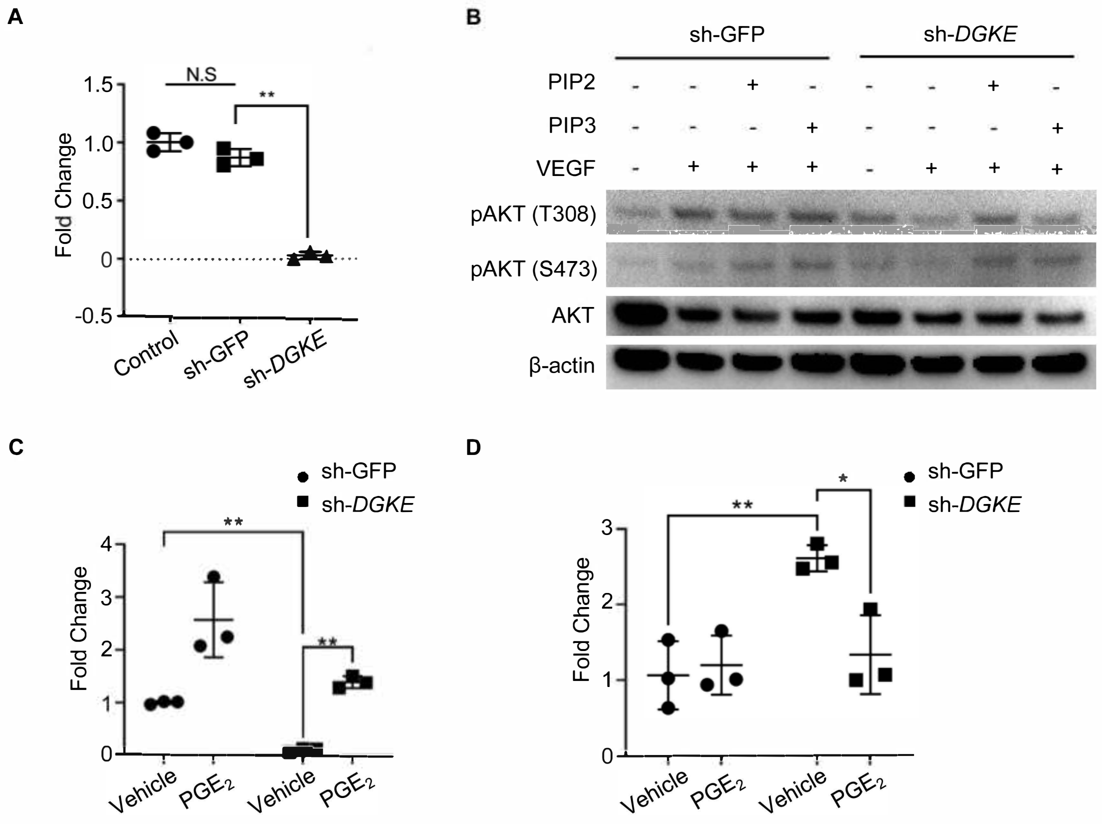

Supplementary figure 2. A. Efficiency of the sh-RNA knockdown in HMECs measured by Q-PCR, compared to non-targeted control (sh-GFP) cells. Controls are non-transfected cells. **B.** Western blots showing that impaired Akt activation in the sh-RNA knockdown HMECs upon VEGFA stimulation is partially reversed by PIP2 and PIP3 supplementation. mRNA expression of **C.** CXCR4 and **D.** MMP-2 in sh-DGKE HMECs and non-target control cells (sh-GFP), with or without PGE2 supplementation. Data are presented as mean ±SD.*: *P*<0.05, **: *P*<0.01(Student's t-test). N.S: non-significant. *n*=3 per group.

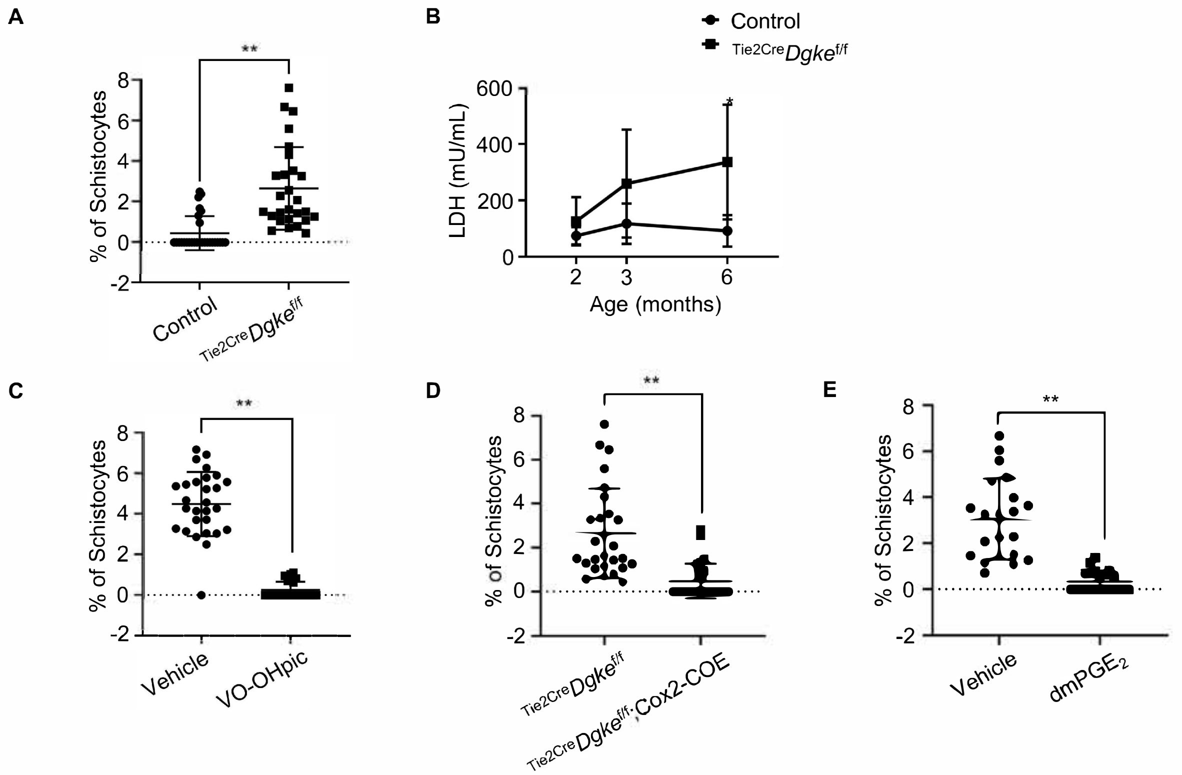

Supplementary figure 3. A. Quantification of the number of circulating schistocytes in Tie2Cre*Dgke*$^{f/f}$ and *Dgke*$^{f/f}$ controls at 6 months of age, expressed as percentage of schistocytes of total number of red blood cells per optical field. **B.** Serum LDH levels in Tie2Cre*Dgke*$^{f/f}$ mice compared to *Dgke*$^{f/f}$ controls at 2, 3, and 6 months of age, *n*=6 mice per group. Percentage of circulating schistocytes in **C.** Tie2Cre*Dgke*$^{f/f}$ mice treated with VO-OHpic or vehicle as a control at 3 months of age; **D.** Tie2Cre*Dgke*$^{f/f}$;Cox2-COE mice and Tie2Cre*Dgke*$^{f/f}$ controls at 3 months of age; **E.** Tie2Cre*Dgke*$^{f/f}$ mice treated with dmPGE$_2$ or vehicle as a control at 3 months of age. Data are presented as mean ±SD.*: *P*<0.05.**: *P*<0.01 (Student's t-test).

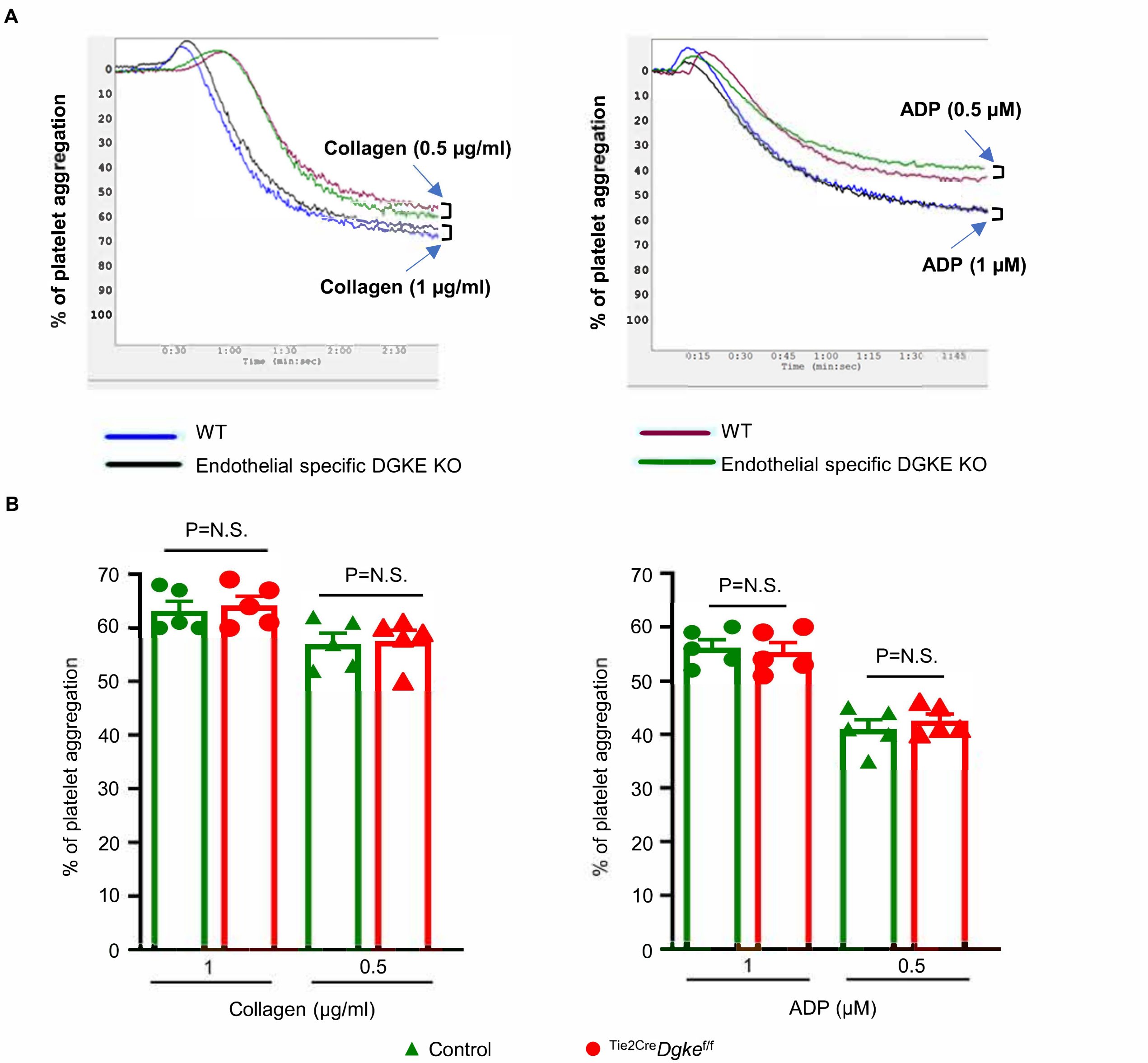

Supplementary figure 4. Aggregation studies of platelets from ^{Tie2Cre}*Dgke*$^{f/f}$ and control mice. A. Representative aggregation curves over time (minutes:seconds) of platelet rich plasmas from ^{Tie2Cre}*Dgke*$^{f/f}$ and control mice, stimulated with two agonists, collagen and adenosine diphosphate (ADP), at sub-optimal and optimal concentrations. Aggregation is expressed as percent of change in light transmission, where 100% refers to transmittance through the blank sample (platelet poor plasma). **B.** Quantification of platelet aggregation from 5 independent experiments, expressed as % of change at maximum amplitude. Data are presented as mean ±SEM. N.S.: non significant (one-way ANOVA followed by Tukey's multiple comparisons test). *n*=5 per group.

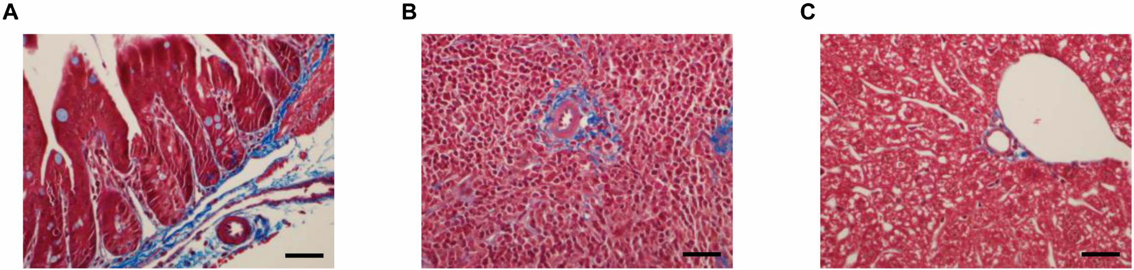

Supplementary figure 5. Representative bright field microscopy images of **A.** small intestine **B.** spleen and **C.** liver of ^{Tie2Cre}*Dgke*$^{f/f}$ conditional knockout mice (H&E stain). No signs of TMA were detected in small arterioles in three mice. Scale bars are 100 mμ.

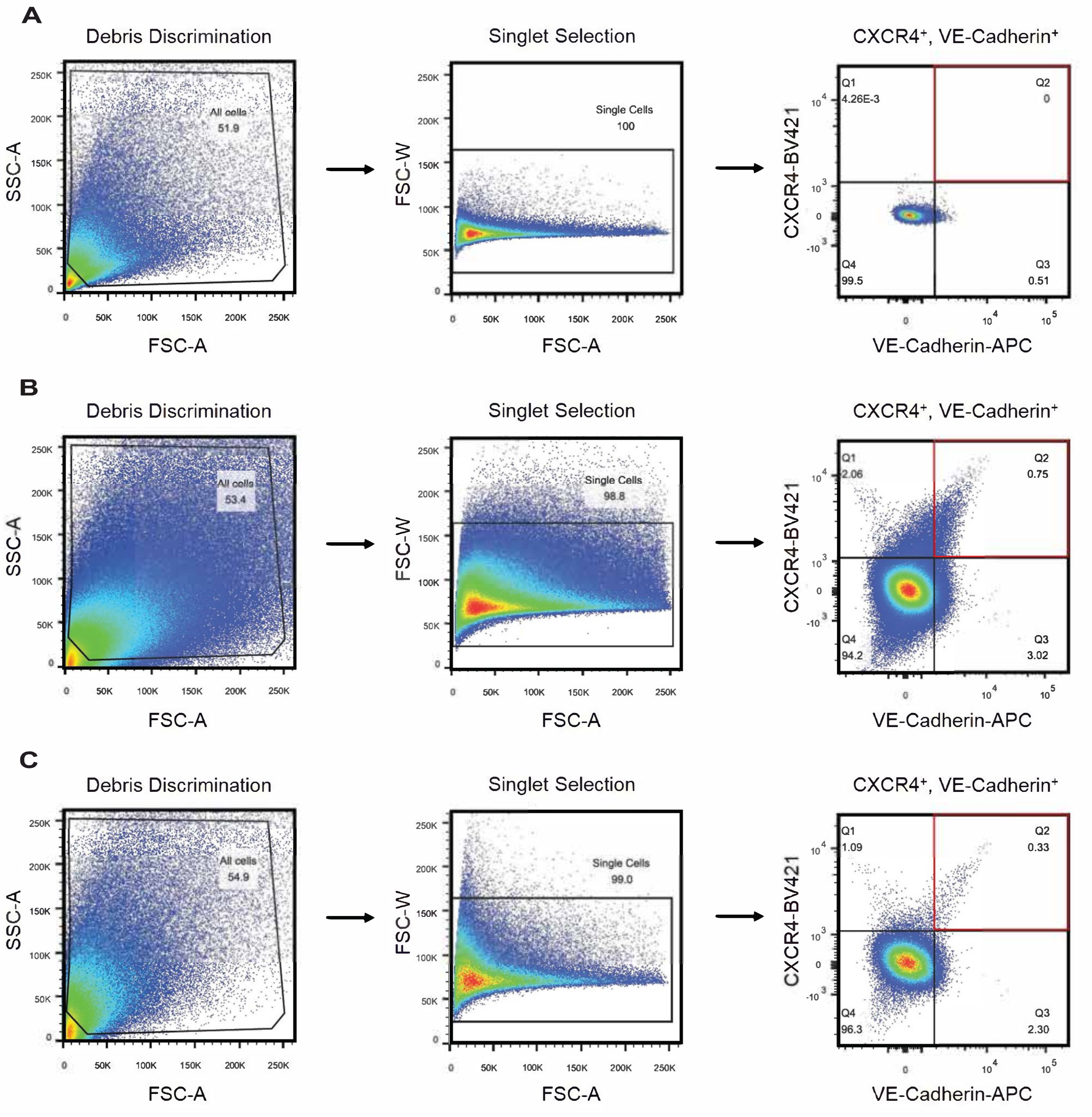

Supplementary figure 6. Flow cytometry gating strategy adopted to generate the data reported in Figure 7, panels F-G and I-J. A. Unstained control (no antibody, cells from control and ^{Tie2Cre}*Dgke*$^{f/f}$ mice, 1:1 ratio). **B.** Control mice, and **C.** ^{Tie2Cre}*Dgke*$^{f/f}$ mice. These experiments were performed in triplicate.

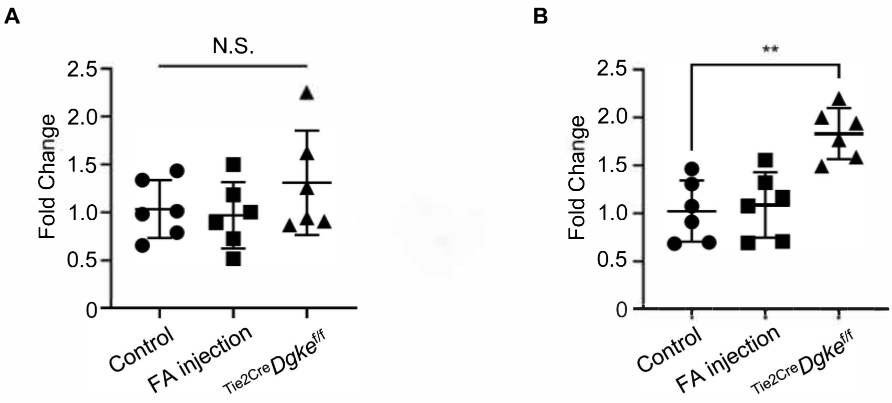

Supplementary figure 7. MMP expression in kidney cortexes. A. MMP9 and **B.** MMP2 expression in control, ^{Tie2Cre}*Dgke*$^{f/f}$, and wildtype mice with Folic Acid (FA) injection. Data are presented as mean ±SD.**: *P*<0.05. N.S.: non significant (Student's t-test). *n*=6 per group.
